# Evidence for transforming growth factor–beta 3 gene polymorphism in non-syndromic cleft lip and palate patients from indian sub-continent

**DOI:** 10.4317/medoral.17453

**Published:** 2011-12-06

**Authors:** Saleem Shaikh, Rajendran Ravenndranath, Moinak Banerjee, Anna Joseph, Pramod Jahgirdar

**Affiliations:** 1 MDS, Reader, Department of Oral and Maxillofacial Pathology, ACPM Dental College, Dhule - Maharashtra; 2 PhD, Professor and Head, (Retd), Department of Oral pathology and Microbiology, Government Dental College, Thiruvananthapuram; 3 PhD, Scientist, Department of Human Molecular Genetics, Rajiv Gandhi Centre for Biotechnology, Thiruvananthapuram; 4 MDS, Reader, Department of Oral pathology, PMS College of Dental Sciences, Vattappara - Thiruvanthapuram; 5 MDS, Reader, Department of Oral pathology, Seema Dental College & Hospital, Rishikesh

## Abstract

Objectives: Orofacial clefts are major human birth defects with complex etiology. Previous studies have proposed Transforming growth factor - beta 3 (TGF-β3) gene as a key player in contributing to non-syndromic cleft lip and palate, however none of the studies have yet included Indian population. Hence this study was designed to detect TGF-β3 gene polymorphism in nonsyndromic cleft lip and palate patients from Indian population which is genetically distinct from previously studied populations. Study Design: Peripheral blood samples of forty non-syndromic cleft lip and palate patients and forty unaffected individuals were collected for a case – control study design. Ethical clearance from the institutional review board and informed consent from all subjects was obtained. DNA extracted from the cases and controls was amplified using polymerase chain reaction (PCR) with TGF-β3 specific primers. The obtained fragments were sequenced and TGF-β3 gene polymorphisms were assessed based on the number of CA repeats. Results: Chi –square test was used to compare the case and control groups. Results showed a significant difference in the number of CA repeats between the case and the control groups (p=0.01). Conclusion: This study confirms the crucial role of TGF-β3 in the fusion of palatal shelves during development and further, provides novel evidence of TGF-β3 gene polymorphism in the etiology of nonsyndromic cleft lip and palate in Indian subpopulation.

** Key words:** Orofacial clefts, nonsyndromic cleft lip with/without cleft palate, TGF-β3, Polymerase chain reaction, gene polymorphism.

## Introduction

Orofacial clefts, and in particular cleft lip and palate, are major human birth defects with complex etiology. Although specific genetic causes for some commonforms of syndromic cleft lip and palate such as Van der Woude syndrome have been identified, the genes for non-syndromic clefting have been more elusive targets (1-3). Many genes have been proposed to play an important role in the causation of cleft lip with/without cleft palate. Transforming Growth Factor-Beta 3 (TGF-β3) is one of the strongest candidate gene for cleft lip and palate in humans (4,5). TGF-β3 (located on 14q24) has a broad spectrum of biological activities and is known to induce palatal fusion (6) and in recent years a large number of studies have been conducted to elucidate the relationship of TGF-β3 and cleft lip and palate (7-9). As none of the studies included patients of the Indian subcontinent who are genetically distinct from the other study populations, this study was done to detect the presence of TGF-β3 gene polymorphisms in non syndromic cleft lip and palate in Indian patients.

## Material and Methods 

 -Patient samples: The study was approved by the Ethics Committee of Medical College Hospital. Forty patients with non-syndromic cleft lip with cleft palate were selected and informed consent was obtained. Subjects with known teratogenic exposure and other recognized syndromes as well as children with other major or multiple minor defects and/or developmental delay as determined from the demographic details, perinatal history, teratogenic exposure and family history were excluded. Forty unaffected individuals from the same geographic area who had no craniofacial anomaly or other congenital disease and no family history of craniofacial malformation were included in the study as controls. Peripheral blood sample (10 ml) was collected by venipuncture from all cases. 

 -DNA extraction and amplification: A modified protocol for standard organic extraction method was used for DNA extraction from lymphocytes. Polymerase chain reactions were performed to amplify the extracted DNA samples and assessed for the allelic variants using specific primers for TGF-β3 gene. 

TGF-β3 primers:

Sense (Forward) primer: AGATTCTGGCTTCCACGAAA 

Antisense (reverse) primer: GCAAGCAGGGATAATAACAGCA.

After completion of the PCR reaction, an agarose gel was run to identify amplified PCR products. These fragments were then cut out and subjected to to sequencing PCR using the ABI prism Big. Dye Terminator Cycle sequencing ready reaction kit version 3.1. The product obtained from sequencing PCR was subjected to isopropanol precipitation to remove protein and other contaminants and then sent for sequencing. 

Analysis of Sequencing Results: The TGF-β3 gene has a CA repeat sequence, and the polymorphism is dependent on the fre-quency of CA repeats. The sequences of the amplified DNA fragments of TGF-β3 gene were analyzed using Bioedit sequence analyzer software. 

 -Statistical procedure used: Chi –square test was used to compare the case and control groups.

## Results

The number of CA repeats in the DNA sequence of the cases was compared with the number of CA repeats in controls; 23 out of a total of 40 samples from the case group showed 10 CA repeats, whereas 14 of them had 9 CA repeats and 3 samples had 6 CA repeats. In the control group 29 out of 40 samples had 9 CA repeats and only 11 had 10 CA repeats ( Table 1). The significance of the results obtained was statistically analyzed using chi-square test. A chi-square value of > 5.99 and a ‘p’ value of <0.05 is considered statistically significant. The chi-square calculated with our data was 12.446 with a corresponding p value of 0.01. Thus, we observed that the difference between the Non-syndromic CLCP cases and the control group in terms of number of CA repeats representing the TGF-β3 polymorphisms is highly significant.

**Table 1:**
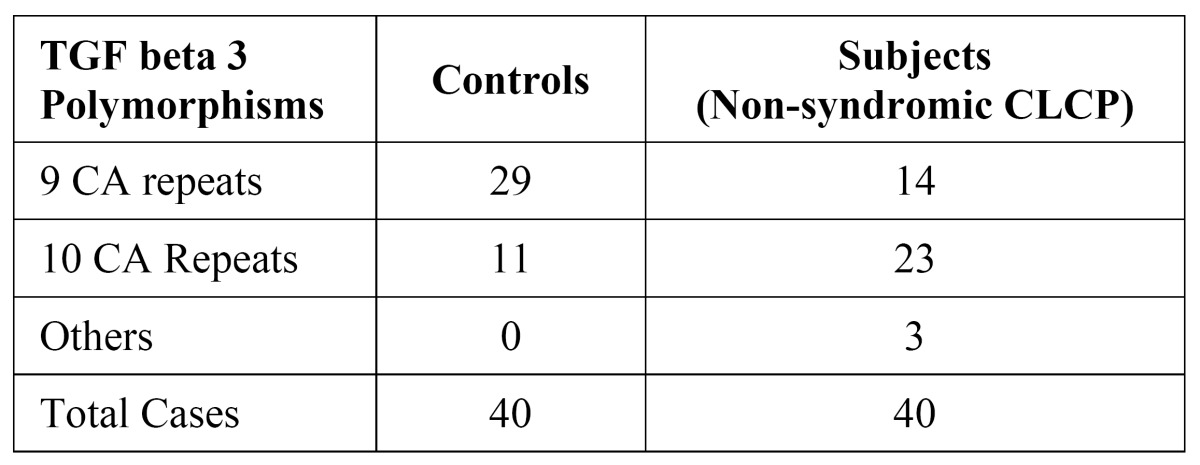
Results of TGF-β3 Polymorphisms as determined by CA repeats in samples.

## Discussion 

The development of orofacial region relies on the interplay of a vast range of genetic and epigenetic factors (10). When these factors are disrupted either at the gene level, due to mutations or by disruption of epigenetic regulation by environmental teratogens could lead to orofacial clefting. Patients with orofacial clefts require surgical, nutritional, dental, speech, medical and behavioral interventions and impose a substantial emotional and economic burden on the society. These issues, along with the relatively high prevalence of orofacial clefts, emphasize the importance of understanding the underlying causes of these defects. Most orofacial clefts are caused by the interaction between genetic and environmental factors (11). Genetic factors create susceptibility for clefts, and when environmental factors (ie, triggers) interact with a genetically susceptible genotype, a cleft develops during an early stage of development (12). The organisation of the facial structures requires tissues to proliferate, fuse and differentiate. Palatogenesis involves fusion of the medial edge epithelium (MEE) of the approximating palatal shelves with each other via numerous desmosome contacts to form a midline palatal seam (13,14). This seam rapidly degenerates allowing mesenchymal cells to flow across the now intact horizontal palate. TGF-β3 is found in the epithelial component of the shelves and in the medial edge epithelium (MEE). TGF-β3 plays a crucial role in these initial adhesive interactions (15). TGF-β3 knock out mouse exhibits cleft palate through failure of palatal shelf fusion (16). Although the palatal shelves otherwise develop normally, they show a marked reduction in the filopodia and show down-regulation of condroitin sulphate proteoglycan on the apical surface of the MEE (17,18), both of which are required for efficient MEE adhesion (19,20). 

A large number of studies (21-24) have been carried out to elucidate the role of TGF-β3 gene polymorphisms in the etiopathogenesis of cleft lip and palate of which some studies, such as that of Vieira AR et al (5) on south American population and Lidral AC et al (22) on Philippine population, have yielded positive associations, while a few studies did not show any significant association, such as those of Tanabe A et al (23) and Jugessur A et al (24) conducted on Japanese and Norwegian population respectively. The prevalence of orofacial clefts is more common among Indians and oriental populations, where it occurs in 1 every 500 births or higher (25). Inspite of this, there has been no major study thus far on Indian patients with non-syndromic CLCP. Our study was, hence, the first report on polymorphism in TGF-β3 gene in patients with non-syndromic cleft lip and palate of Indian origin. This study could provide a basis for more elaborate studies involving larger number of samples that can explore the specific role of TGF-β3gene in more detail in this population. 

The general failure to pinpoint the precise molecular events that lead to human cleft lip and palate most likely stems from our lack of knowledge about the gene networks and regulation of gene expression during palatal development. While studies such as ours are related to individual gene events, they could help by contributing to the existing scientific knowledge of inter-related gene networks, ultimately leading to a better understanding of the pathogenesis of cleft lip and palate. In addition to playing an important role in the development of palate, TGF-β3 also has some therapeutic possibilities: exogenous TGF3 can correct the palatal fusion defect in TGF-β3-null embryos, raising the possibility of exogenous supplementation of TGF-β3 as fetal therapy. Rather than a surgical approach, it is known that maternally-administered recombinant TGF-β3 can cross the mouse placental barrier. Exogenous TGF-β3 is also known to reduce the severity of scarring following wound healing in rats (26), raising the possibility that some individuals with cleft palate caused by genetic abnormalities in the TGF-β3 pathway might be more prone to excessive scarring following surgical correction, thereby compounding an otherwise distressing abnormality. Exogenous application of TGF-β3 as an anti-scarring therapy at the time of surgical correction of the cleft may also be particularly beneficial to such individuals. 

In summary, our study highlights the role for TGF-β3 in non-syndromic cleft lip and palate patients from the Indian sub-continent and points out a potential therapeutic intervention to correct the malady. 

## References

[1] Mitchell LE, Christensen K (1996). Analysis of the recurrence patterns for nonsyndromic cleft lip with or without cleft palate in the families of 3,073 Danish probands. Am J Med Genet.

[2] Hecht JT, Yang P, Michels VV, Buektow KH (1991). Complex segregation analysis of nonsyndromic cleft lip and palate. Am J Hum Genet.

[3] Wong FK, Hagberg C, Karsten A, Larson O, Gustavsson M, Huggare J (2000). Linkage analysis of candidate regions in Swedish nonsyndromic cleft lip with or without cleft palate families. Cleft Palate Craniofac J.

[4] Brunet CL, Sharpe PM, Ferguson MW (1995). Inhibition of TGF-beta 3 (but not TGF-beta 1 or TGF-beta 2) activity prevents normal mouse embryonic palate fusion. Int J Dev Biol.

[5] Vieira AR, Orioli IM, Castilla EE, Cooper ME, Marazita ML, Murray JC (2003). MSX1 and TGFB3 contribute to clefting in South America. J Dent Res.

[6] Dudas M, Nagy A, Laping NJ, Moustakas A, Kaartinen V (2004). Tgf-beta3-induced palatal fusion is mediated by Alk-5/Smad pathway. Dev Biol.

[7] Kim MH, Kim HJ, Choi JY, Nahm DS (2003). Transforming growth factor–beta 3 gene SfaN1 polymorphism in Korean nonsyndromic cleft lip and palate patients. J Biochem Mol Biol.

[8] Sun D, Vanderburg CR, Odierna GS, Hay ED (1998). TGFbeta3 promotes transformation of chicken palate medial edge epithelium to mesenchyme in vitro. Development.

[9] Taya Y, O’Kane S, Ferguson MW (1999). Pathogenesis of cleft palate in TGF-beta3 knockout mice. Development.

[10] Murray JC (1995). Face facts: genes, environment, and clefts. Am J Hum Genet.

[11] Fraser FC (1970). The genetics of cleft lip and cleft palate. Am J Hum Genet.

[12] Schutte BC, Murray JC (1999). The many faces and factors of orofacial clefts. Hum Mol Genet.

[13] Ferguson MW (1988). Palate development. Development.

[14] Ferguson WJ (1984). Epithelial-mesenchymal interactions during vertebrate palatogenesis. Curr Top Dev Biol.

[15] Kaartinen V, Cui XM, Heisterkamp N, Groffen J, Shuler CF (1997). Transforming growth factor-beta3 regulates transdifferentiation of medial edge epithelium during palatal fusion and associated degradation of the basement membrane. Dev Dyn.

[16] Tudela C, Formoso MA, Martínez T, Pérez R, Aparicio M, Maestro C (2002). TGF-beta3 is required for the adhesion and intercalation of medial edge epithelial cells during palate fusion. Int J Dev Biol.

[17] Martinez-Alvarez C, Tudela C, Perez-Miguelsanz J, O’Kane S, Puerta J, Ferguson MW (2000). Medial edge epithelial cell fate during palatal fusion. Dev Biol.

[18] Kohama K, Nonaka K, Hosokawa R, Shum L, Ohishi M (2002). TGF-beta-3 promotes scarless repair of cleft lip in mouse fetuses. J Dent Res.

[19] Proetzel G, Pawlowski SA, Wiles MV, Yin M, Boivin GP, Howles PN (1995). Transforming growth factor-beta 3 is required for secondary palate fusion. Nat Genet.

[20] Tyler MS, Koch WE (1977). In vitro development of palatal tissues from embryonic mice III Interactions between palatal epithelium and heterotypic oral mesenchyme. J Embryol Exp Morphol.

[21] Baroni T, Carinci P, Bellucci C, Lilli C, Becchetti E, Carinci F (2003). Cross-talk between interleukin-6 and transforming growth factor-beta3 regulates extracellular matrix production by human fibroblasts from subjects with non-syndromic cleft lip and palate. J Periodontol.

[22] Lidral AC, Murray JC, Buetow KH, Basart AM, Schearer H, Shiang R (1997). Studies of the candidate genes TGFB2, MSX1, TGFA, and TGFB3 in the etiology of cleft lip and palate in the Philippines. Cleft Palate Craniofac J.

[23] Tanabe A, Taketani S, Endo-Ichikawa Y, Tokunaga R, Ogawa Y, Hiramoto M (2000). Analysis of the candidate genes responsible for non-syndromic cleft lip and palate in Japanese people. Clin Sci (Lond).

[24] Jugessur A, Lie RT, Wilcox AJ, Murray JC, Taylor JA, Saugstad OD (2003). Variants of developmental genes (TGFA, TGFB3, and MSX1) and their associations with orofacial clefts: a case-parent triad analysis. Genet Epidemiol.

[25] Hagberg C, Larson O, Milerad J (1998). Incidence of cleft lip and palate and risks of additional malformations. Cleft Palate Craniofac J.

[26] Shah M, Foreman DM, Ferguson MW (1995). Neutralisation of TGF-beta 1 and TGF-beta 2 or exogenous addition of TGF-beta 3 to cutaneous rat wounds reduces scarring. J Cell Sci.

